# Early Complications of Planned Resection Versus Unplanned Excision of Sarcomas in the Distal Upper Extremity

**DOI:** 10.1016/j.jhsg.2024.04.010

**Published:** 2024-05-18

**Authors:** Seth Ahlquist, Kevin Y. Chen, Eric Chang, Scott D. Nelson, Nicholas M. Bernthal, Lauren E. Wessel

**Affiliations:** ∗ Department of Orthopaedic Surgery, David Geffen School of Medicine at UCLA, Los Angeles, CA

**Keywords:** Sarcoma, Unplanned excision, Upper extremity

## Abstract

**Purpose:**

Unplanned excisions are defined as excisions of malignant tumors performed without preoperative cross-sectional imaging or diagnostic biopsy, frequently resulting in residual disease and re-excision secondary to positive surgical margins. The purpose of this study was to compare the relative morbidity of planned versus unplanned upper-extremity sarcoma excisions.

**Methods:**

A single tertiary referral hospital pathology database was queried from January 2015 through 2022 for primary upper-extremity sarcomas (forearm, wrist, hand, and finger). Demographics, tumor features, survival characteristics, and outcomes were retrospectively reviewed.

**Results:**

Forty-two upper-extremity sarcoma patients were identified, two-thirds of whom had unplanned excisions. Those with unplanned excisions were more likely to be female (relative risk [RR]: 1.9; *P* = .002), undergo initial excision at a nonsarcoma center (RR: 14.0; *P* < .001), have masses distal to the forearm (RR: 1.6; *P* = .02), and have smaller masses (4.8 vs 7.4 cm, *P* = .03). 71.4% of tumors were high grade, and 60.7% less than 5 cm in size.

Unplanned excisions had positive margins in 96.4% of cases and were more likely to undergo re-excision (odds ratio [OR]: 20.0; *P* = .001), more total resections (2.7 vs 1.4, *P* = .009), sacrifice of neurovascular structures (OR: 6.1; *P* = .04), adjuvant radiation therapy (OR: 4.5; *P* = .05), adjuvant systemic therapy (OR: 10.9; *P* = .03), or experience a complication (OR: 17.6; *P* = .002) at an average of 38.0 months of follow-up.

Nearly half of all unplanned excision patients developed a local recurrence or metastatic disease. Six patients required an amputation versus one in the planned cohort (*P* = .17), and 26.5% of patients died at an average of 32.5 months from presentation.

**Conclusions:**

Distal upper-extremity sarcoma excisions are frequently unplanned, with high rates of morbidity compared with planned excisions. Surgeons should have a low threshold for cross-sectional imaging and core needle biopsy of atypical lesions, irrespective of size, with referral to a sarcoma center.

**Type of study/level of evidence:**

Prognostic IV.

Soft tissue sarcomas (STS) are rare, accounting for approximately two cases per million people annually, and are even less common in the upper extremity.^1^, ^2^, ^3^ Nevertheless, these tumors continue to have poor prognosis with a 5-year survival rate of around 62%.^4^ Possibly owing to their low prevalence, a considerable proportion of STS are inadvertently excised without prior oncologic work-up. When performed without a preoperative understanding of malignancy or relevant imaging and staging work-up, these procedures are considered “unplanned excisions,” with rates reported in the literature ranging from 18% to 83% among patients with STS.^5^, ^6^, ^7^, ^8^, ^9^, ^10^, ^11^, ^12^, ^13^, ^14^ There are conflicting data on whether unplanned sarcoma excisions impact long-term outcomes and whether upper-extremity STS have the same prognosis/behavior as lesions in other locations.^2^^,^^3^^,^^7^^,^^8^^,^^10^^,^^15^, ^16^, ^17^, ^18^, ^19^, ^20^

Unplanned excisions can often be associated with residual tumors and the need for re-excision to achieve negative margins.^9^ Upper-extremity sarcomas pose a particularly challenging management problem because of the complex anatomy and proximity to critical neurovascular structures. In such circumstances, aggressive resections or reresections may have considerable implications for hand function. Prior studies have examined the outcomes of unplanned excisions generally and in the extremities, with mixed results, but few have studied the outcomes in the upper extremity.^5^, ^6^, ^7^, ^8^, ^9^, ^10^^,^^12^, ^13^, ^14^, ^15^, ^16^, ^17^^,^^21^, ^22^, ^23^, ^24^, ^25^, ^26^, ^27^, ^28^

The purpose of this study was to evaluate early complications of unplanned sarcoma excisions compared with planned resection in the upper extremity. Our hypothesis was that unplanned upper-extremity sarcoma surgeries would be associated with worse oncologic and functional outcomes when compared with those of patients with planned resections.

## Methods

A retrospective review of sarcoma patients at The University of California, Los Angeles was used to identify all patients with a distal upper-extremity biopsy (forearm, wrist, hand, and finger) from January 1, 2015 to January 1, 2022 in the institutional pathology database. The forearm was defined as distal to the elbow joint. Approval for this single-institution study was obtained from the institutional review board. After patients with dermatologic lesions were identified and excluded, 7,888 pathology reports were reviewed for a diagnosis of sarcoma, which yielded 119 entries that corresponded to 74 patients. Forty-two patients who proceeded with clinical care at our institution were identified. The remainder was noted solely for pathology consultation. The demographics of patients not included in the study did not significantly differ from those who were included in the study. Surgical reports, clinic notes, pathology reports, and imaging studies were reviewed for each patient. Baseline demographic variables including age, sex (as biologically assigned at birth), and race were collected for all patients in addition to tumor-specific characteristics of location, tissue type, pathologic diagnosis, grade, and size. As a referral center, many patients present for surgery and may follow-up with a local sarcoma medical oncologist. Oncologic outcomes were collected, including margin status, number of excisions, recurrence, metastasis, complications, mortality and need for soft tissue coverage, adjuvant therapy, and amputation for those patients with a minimum of 6-month follow-up at our institution (eight patients being excluded). Tumor size was defined as the longest documented length in a single dimension from preoperative cross-sectional imaging. If there was no preoperative imaging available in the setting of an unplanned excision, the pathology report was used to define the dimension. Radiographic dimension was favored, if available, to exclude possible cytoreductive effects in the setting of radiation or systemic therapy (chemotherapy vs immunotherapy). Seven surgeons provided surgical and postsurgical care to patients (four orthopedic oncology surgeons and three general surgical oncologists).

Categorical variables are reported as frequencies and percentages, whereas continuous variables are presented as means with standard deviations. Chi-square tests were performed to establish significant differences between groups. Odds ratios and relative risk ratios were calculated to delineate the magnitude of this difference. Relative risk ratios are reported when odds ratios are unable to be calculated because of a cohort having zero patients with a given outcome measure. A two-tailed *P* value of .05 was considered significant for all tests. This study adhered to the Strengthening the Reporting of Observational Studies in Epidemiology guidelines.

## Results

Forty-two patients were identified (14 planned and 28 unplanned). The overall cohort comprised 69.0% men, 69.0% White, with a mean age of 55.7 ± 22.1 years and an average follow-up of 31.2 ± 30.5 months (Table 1). Moreover, 73.8% of sarcomas were in the forearm, 95.2% were of soft tissue in origin, and 71.4% were high grade, and the greatest dimension was 5.7 ± 3.5 cm on average. Symptom duration prior to treatment was 10.1 ± 16.4 months. The most common pathologic diagnoses were undifferentiated pleiomorphic sarcoma (35.6%), myxofibrosarcoma (14.3%), and either epithelioid sarcoma or synovial sarcoma (9.5% each).

**Table 1:** Planned Versus Unplanned Upper-Extremity Sarcoma Excision Cohort Characteristics

Variable	Planned n = 14 (%)	Unplanned n = 28 (%)	Relative Risk (95% CI)	*P*
Cohort characteristic				
Age (y)	50.8 ± 27.7	58.1 ± 19.3		.32
Sex				
Male	14 (100.0)	15 (53.6)	1.9 (1.3–2.6)	.002
Female	0	13 (46.4)		
Race				
White	7 (50.0)	22 (78.6)		.09
Asian	5 (35.7)	4 (14.3)		
Hispanic	1 (7.1)	2 (7.1)		
Black	1 (7.1)	0		
Anatomical location				
Forearm	14 (100)	18 (64.3)	1.6 (1.2–2.1)	.02
Wrist	0	3 (10.7)		
Hand	0	5 (17.9)		
Finger	0	2 (7.1)		
Mass tissue type				
Soft tissue	13 (92.9)	27 (96.4)		1.0
Bone	1 (7.1)	1 (3.4)		
Symptom duration	11.3 mo	9.5 mo		.76
Initial excision facility				
Outside facility	0	26 (92.9)	14.0 (3.7–53.2)	< .001
Sarcoma center	14 (100.0)	2 (7.1)		
Pathologic diagnosis				
UPS	3 (21.4)	12 (42.9)		.33
Epithelioid sarcoma	0	4 (14.3)		
Myxofibrosarcoma	3 (21.4)	3 (10.7)		
Rhabdomyosarcoma	2 (14.3)	0		
Synovial sarcoma	1 (7.1)	3 (10.7)		
Leiomyosarcoma	1 (7.1)	2 (7.1)		
Dermatofibrosarcoma	1 (7.1)	1 (3.6)		
Chondrosarcoma	1 (7.1)	1 (3.6)		
Liposarcoma	1 (7.1)	1 (3.6)		
Fibrosarcoma	1 (7.1)	0		
OFM sarcoma	0	1 (3.4)		
Grade				
High	10 (71.4)	20 (71.4)		.12
Intermediate	0	5 (17.9)		
Low	4 (28.6)	3 (10.7)		
Size				
<5 cm	5 (35.7)	17 (60.7)		.19
Average	7.4 ± 4.5 cm	4.8 ± 2.7 cm		.03
Positive margins	3 (21.4)	27 (96.4)	22.0 (3.1–153.7)	< .001
Follow-up	35.4 mo	29.1 mo		.27

OFM, ossifying fibromyxoid; UPS, undifferentiated pleiomorphic sarcoma.

The unplanned excision cohort was more likely to be women (46.4% vs 0%, RR: 1.9, 95% confidence interval [CI]: 1.3–2.6, *P* = .002), undergo initial surgery at a nonsarcoma center (92.9% vs 0%; RR: 14.0, 95% CI: 3.7–53.2, *P* < .001), have a mass distal to the forearm (wrist, hand, and fingers) (RR: 1.6, 95% CI: 1.2–2.1, *P* = .02), present with a smaller mass (4.8 vs 7.4 cm, *P* = .03), and have a positive margin after the initial procedure (96.4% vs 21.4%, RR: 22.0, 95% CI: 3.1–153.7, *P* < .001). There were no significant differences between the planned and unplanned excision cohorts in terms of age, race, mass tissue type, pathologic diagnosis distribution, tumor grade, and duration of pretreatment symptoms or follow-up (Table 1).

The unplanned excision cohort was more likely to undergo re-excision (85.7% vs 23.1%, OR: 20.0, 95% CI: 3.4–118.3, *P* =.001), more total surgeries for resection (2.7 vs 1.4, *P* < .001), sacrifice of neurovascular structures (52.4% vs 15.4%, OR: 6.1, 95% CI: 1.1–34.2, *P* = .04), adjuvant radiation therapy (66.7% vs 30.8%, OR: 4.5, 95% CI: 1.0–19.9, *P* = .05), adjuvant systemic therapy (47.6% vs 7.7%, OR: 10.9, 95% CI: 1.2–99.7, *P* = .03), or experience a complication (76.2% vs 15.4%, OR: 17.6, 95% CI: 2.9–107.6, *P* = .002) (Table 2).

**Table 2:** Planned Versus Unplanned Upper-Extremity Sarcoma Excision Outcomes

Variable	Planned n = 13 (%)	Unplanned n = 21 (%)	Odds Ratio (95% CI)	*P*
Outcome				
Re-excision	3 (23.1)	18 (85.7)	20.0 (3.4–118.3)	**.001**
Total re-excisions	0.3 ± 0.5	1.0 ± 0.5		**< .001**
Total excisions	1.4 ± 0.9	2.7 ± 1.5		**.009**
Local recurrence	4 (30.8)	10 (47.6)		.34
Total local recurrences	0.4 ± 0.7	0.9 ± 1.2		.20
NV structures sacrificed	2 (15.4)	11 (52.4)	6.1 (1.1–34.2)	**.04**
Skin graft (split thickness)	1 (7.7)	3 (14.3)		.57
Flap∗	4 (30.8)	9 (42.9)		.48
Amputation†	1 (7.7)	6 (28.6)		.17
Complications‡	2 (15.4)	16 (76.2)	17.6 (2.9–107.6)	**.002**
Metastatic disease	6 (46.1)	9 (42.9)		.85
Adjuvant XRT	4 (30.8)	14 (66.7)	4.5 (1.0–19.9)	**.05**
Adjuvant systemic therapy	1 (7.7)	10 (47.6)	10.9 (1.2–99.7)	**.03**
Mortality	5 (38.5)	4 (19.0)		.28

ALT, anterolateral thigh; NV, neurovascular; PIN, posterior interosseus nerve; PNA, pneumonia; PTX, pneumothorax; SSI, surgical site infection; UTI, urinary tract infection; XRT, radiation therapy.

∗ Advancement flap (8), triceps flap (3), ALT free flap (1), randomized pedicle flap (1).

† Transhumeral (3), below elbow (1), double ray amputation (2), thumb disarticulation (1).

‡ Wound dehiscence (3), SSI (3), dysesthesia/phantom limb (3), PTX (2), seroma (2), infected hematoma (1), UTI (1), brachial artery pseudoaneurysm (1), PNA (1), PIN Nerve Palsy (1).

Approximately half of the unplanned excision patients developed a local recurrence, developed metastatic disease, or required flap coverage after resection, but these outcomes were not significantly more likely to occur than in the planned excision cohort (Table 2). Six patients underwent amputation in the unplanned excision group versus only one in the planned excision cohort (28.6 vs 7.7%, *P* = .17). Mortality was also not significantly different between the cohorts.

## Discussion

Upper-extremity STS present a formidable management challenge, in that a considerable proportion of patients undergo unplanned excision without standard oncologic work-up. In this series, we identified increased morbidity among patients who underwent unplanned STS excision in comparison to a cohort of patients who did not.

Unplanned excisions constituted 66% of our study cohort (Table 1), which aligns with prior reports ranging from 18% to 83%.^5^, ^6^, ^7^, ^8^, ^9^, ^10^, ^11^, ^12^, ^13^, ^14^^,^^23^ The increased proportion of unplanned sarcoma excisions in our study may be due to our institution being one of the highest volume sarcoma referral centers in the country. Several studies have directly compared the proportion of unplanned excisions in the lower extremity (30.8% to 46.9%) with that in the upper extremity and have found a higher proportion in the upper extremity (49.0% to 64.0%).^8^^,^^13^^,^^20^^,^^29^ This phenomenon may be attributable to their small size, often painless nature, and the high prevalence of benign lesions in this region.

Of concern with unplanned excisions is the potential for residual tumors, leading to persistent microscopic disease, local recurrence, metastasis, and eventual disease-related mortality. The risk of residual disease in the setting of unplanned excisions has been demonstrated in the literature.^5^^,^^9^ However, there is a lack of consensus regarding its impact on local recurrence and metastasis rates, with some studies reporting an elevated risk and others suggesting that these rates are similar to that of planned excisions.^3^^,^^12^, ^13^, ^14^^,^^22^ Although our study did not find an increased rate of local recurrence or metastases, this may be explained by the relatively short follow-up and limited sample size of our cohorts (Table 1).

Our results align with those of previous research indicating that unplanned excision results in more surgeries/re-excisions as well as adjuvant therapy.^8^^,^^22^^,^^23^ In our study, the use of adjuvant radiation or systemic therapy was predominantly dictated by margin status as opposed to the identity of the tumor subtype. Notably, our unplanned excision cohort also had a higher rate of neurovascular structure resection and complications (Table 2), which have considerable ramifications for patients. Neurovascular resections were frequently required because of contamination during incomplete initial excision, although also at other times were unavoidable because of direct involvement of the tumor with these structures. Surgical complications that have previously been shown to be more common in unplanned sarcoma excisions but conspicuously not so in our cohort were amputations and soft tissue reconstructions.^12^^,^^14^^,^^21^, ^22^, ^23^ In our study, amputations were more common in the unplanned excision cohort (six vs one in the planned cohort). Although this did not reach statistical significance, we believe these results are limited by sample size but remain clinically relevant in their reflection of uncertainty in the location of residual disease after unplanned excision. In such clinical situations, orthopedic oncologists may often be more likely to recommend amputation for local control. Soft tissue reconstructions were not more common in the unplanned excision cohort in our study, which may be explained by planned excisions involving a larger overall mass at initial presentation, requiring more extensive resection, and a similar rate of subsequent flap reconstruction to the unplanned cohort (30.8% vs 42.9%, *P* = .48, Table 2).

In the present study, planned excisions had a nonsignificantly higher mortality rate (Table 2). Although Rougraff et al^16^ showed lower survival in unplanned excisions in masses greater than 4 cm, other authors have reported similar oncologic outcomes between planned and unplanned excisions.^7^^,^^8^^,^^10^^,^^14^^,^^15^^,^^23^ Although our study was limited by sample size and referral bias, larger prospective studies to evaluate this question specifically would be of major value in understanding differences in survival.

Notable risk factors for unplanned excision in our study included female sex, tumor location distal to the forearm, excision at a nonsarcoma center, and smaller average mass size (Table 1). Conversely, Smolle et al^8^ reported a higher likelihood of unplanned excision in men, although their study also included sarcomas in the lower extremity and trunk. Although the literature lacks a direct comparison of unplanned excision frequencies among different upper-extremity locations, our results are supported by studies reporting an 83% rate of hand sarcomas versus 63% in those proximal to the wrist/hand.^20^^,^^23^

There are mixed results as to whether size is a risk factor for unplanned sarcoma excision, with two studies reporting a high risk in larger masses and one reporting a higher risk in smaller masses, with the latter study corresponding with our findings.^8^^,^^14^^,^^23^ There has been a longstanding focus on developing biopsy indication guidelines, with several recommending biopsy for masses larger than 5 cm.^30^, ^31^, ^32^ This is notable, given the average size of unplanned sarcoma excisions in our study was 4.8 cm, suggesting that the current size threshold guideline may not be applicable to upper-extremity tumors, which present at smaller sizes while remaining high grade (Table 1). This must also be taken in context that most upper-extremity masses less than 5 cm are benign, and therefore, it may be impractical to image/biopsy all such masses.^30^, ^31^, ^32^

The present study has limitations. As a tertiary center, cases are referred from hospitals in distant locations with fewer resources. These patients frequently have surgery and follow-up at our institution and then subsequently continue with surveillance follow-up at their home institution with a medical or radiation oncologist, decreasing the duration of our follow-up. As a result, we may be underreporting rates of local recurrence, metastasis, and complications; however, we required a 6-month follow-up at our institution for inclusion in this analysis. Additionally, given that multiple surgeons are included in the study, the results may be confounded by individual surgeon characteristics. Notably, however, all cases were discussed with the same multidisciplinary tumor board for treatment planning; hence, surgical indications were likely similar among surgeons. As a retrospective study, there is also possible selection bias where only the most complicated planned excision as well as positive margin unplanned excision cases are referred to our institution, which may affect the complications and times for return to the operating room in each group. There are also differences in the fact that the cohorts are nonmatched in terms of baseline demographics. A large prospective, multicenter study would be invaluable in collecting these data in a meaningful volume in a prospective fashion.

The results of the present study suggest that unplanned excisions have higher rates of early complication compared with those with appropriate oncologic work-up, including increased surgeries/re-excisions, neurovascular structure resections, complications, and need for adjuvant radiation and chemotherapy. Previous biopsy guidelines may not be applicable to upper-extremity tumors, given the average dimension of unplanned tumor excisions in our study was less than 5 cm while frequently remaining high grade. Risk factors for unplanned excision and poorer oncologic outcomes may include female sex, location distal to the forearm, and smaller size. Surgeons should have a low threshold for cross-sectional advanced diagnostic imaging and core needle biopsy concerning upper extremity masses irrespective of whether these meet the generally published guidelines, with referral to a sarcoma center for definitive treatment.

## Conflicts of Interest

No benefits in any form have been received or will be received related directly to this article.
